# Surveillance-response systems: the key to elimination of tropical diseases

**DOI:** 10.1186/2049-9957-3-17

**Published:** 2014-05-27

**Authors:** Ernest Tambo, Lin Ai, Xia Zhou, Jun-Hu Chen, Wei Hu, Robert Bergquist, Jia-Gang Guo, Jürg Utzinger, Marcel Tanner, Xiao-Nong Zhou

**Affiliations:** 1 National Institute of Parasitic Diseases, Chinese Center for Disease Control and Prevention, Shanghai 200025, People’s Republic of China; 2 Biochemistry Department, Center for Sustainable Malaria Control, Faculty of Natural & Agricultural Sciences, University of Pretoria, Hatfield 0028, South Africa; 3 WHO Collaborating Centre for Malaria, Schistosomiasis and Filariasis, Key Laboratory of Parasite and Vector Biology, Ministry of Health, Shanghai 200025, People’s Republic of China; 4 Sochow University, Suzhou 215123, People’s Republic of China; 5 School of Life Science, Fudan University, Shanghai 200433, People’s Republic of China; 6 Ingerod 407, Brastad, Sweden; 7 Department of Control of Neglected Tropical Diseases, World Health Organization, Avenue Appia 20, CH-1211 Geneva, Switzerland; 8 Department of Epidemiology and Public Health, Swiss Tropical and Public Health Institute, P.O. Box, CH-4002 Basel, Switzerland; 9 University of Basel, P.O. Box, CH-4003 Basel, Switzerland

**Keywords:** Tropical diseases, Control, Elimination, Surveillance-response system, Global health, China

## Abstract

Tropical diseases remain a major cause of morbidity and mortality in developing countries. Although combined health efforts brought about significant improvements over the past 20 years, communities in resource-constrained settings lack the means of strengthening their environment in directions that would provide less favourable conditions for pathogens. Still, the impact of infectious diseases is declining worldwide along with progress made regarding responses to basic health problems and improving health services delivery to the most vulnerable populations. The London Declaration on Neglected Tropical Diseases (NTDs), initiated by the World Health Organization’s NTD roadmap, set out the path towards control and eventual elimination of several tropical diseases by 2020, providing an impetus for local and regional disease elimination programmes. Tropical diseases are often patchy and erratic, and there are differing priorities in resources-limited and endemic countries at various levels of their public health systems. In order to identify and prioritize strategic research on elimination of tropical diseases, the ‘First Forum on Surveillance-Response System Leading to Tropical Diseases Elimination’ was convened in Shanghai in June 2012. Current strategies and the NTD roadmap were reviewed, followed by discussions on how to identify and critically examine prevailing challenges and opportunities, including inter-sectoral collaboration and approaches for elimination of several infectious, tropical diseases. A priority research agenda within a ‘One Health-One World’ frame of global health was developed, including (i) the establishment of a platform for resource-sharing and effective surveillance-response systems for Asia Pacific and Africa with an initial focus on elimination of lymphatic filariasis, malaria and schistosomiasis; (ii) development of new strategies, tools and approaches, such as improved diagnostics and antimalarial therapies; (iii) rigorous validation of surveillance-response systems; and (iv) designing pilot studies to transfer Chinese experiences of successful surveillance-response systems to endemic countries with limited resources.

## Multilingual abstracts

Please see Additional file 1 for translations of the abstract into the six official working languages of the United Nations.

## Background

Effective and timely surveillance and responses tailored to specific settings reflect the ability of a health system to provide reliable and judicious information for public health policy, pragmatic action in infectious disease control and elimination, and efficient sustainable development [1,2]. Tropical diseases, including the neglected tropical diseases (NTDs), comprise a group of infectious diseases primarily affecting the poorest segments of society in the tropics and subtropics [3-5]. Although the NTDs affect more than a billion people in the world, the research community, policy makers, implementers and other stakeholders have only recently embarked on assessing and elucidating the micro-epidemiology involved in the eventual elimination of these diseases [3,6-9]. The smallpox and polio eradication programmes provide convincing examples of the critical role played by a globally organised approach in linking surveillance data to targeted, swift and effective public health responses [10-12].

Despite growing international funding and logistical support offered by the World Health Organization (WHO), multilateral agencies, philanthropic organisations and a host of new consortia to fight tropical diseases, the public health burden and the challenges facing programmes to achieve sustained control and ultimate elimination of the major NTDs are still enormous [13,14]. It has been argued that effective surveillance-response systems are a key feature of reliable information on the prevalence, incidence, spatio-temporal distribution and burden of diseases, which, in turn, is essential for prevention, control and elimination [5]. Rapid responses to outbreaks and emergencies are critical to ascertain the effective realization of targets, as well as consolidation of current achievements towards meeting the Millennium Development Goals and the post-2015 agenda of sustainable development [9,15,16]. Operational research defining surveillance-response strategies based on local and contextual situations is urgently needed to identify and validate suitable research priorities with respect to elimination and eradication scenarios [17-22].

The ‘First Forum on Surveillance-Response System Leading to Tropical Disease Elimination’ was held on June 16-18, 2012 in Shanghai. The Forum was jointly supported by the National Institute of Parasitic Diseases of the Chinese Center for Disease Control and Prevention (NIPD/China CDC), the Swiss Tropical and Public Health Institute (Swiss TPH) and the WHO. The purpose was to share knowledge and experiences pertaining to the control, prevention and elimination of major tropical diseases and to discuss novel approaches towards the establishment of integrated surveillance-response platforms and networks to assist these efforts. The Forum brought together scientists, public health experts and health policy specialists to explore and discuss promising surveillance-response systems with focus on, but not limited to, malaria, schistosomiasis, lymphatic filariasis and other NTDs. The participants in each session were engaged in in-depth discussion on current challenges, opportunities and measures to foster reliable effective surveillance-response systems as the strategic key for endemic countries moving towards disease elimination.

It was felt that the establishment of a conceptual framework to design and implement surveillance-response systems is critical for the elimination of tropical diseases including NTDs. Surveillance-response systems with regard to infectious diseases of poverty have gained traction, particularly in settings where health systems provide an integrated data repository capable of characterising disease dynamics at multiple scales. Evaluating the impact of implemented programme(s) and intervention(s) in public health centres and health services on the population-level towards prevention or control of tropical diseases would benefit by a system for surveillance and response leading towards maximum positive health outcomes. To summarise, the two-fold objectives of this paper were: (i) to establish a platform for resource-sharing and effective surveillance-response systems for Asia Pacific and Africa with an initial focus on elimination of lymphatic filariasis, malaria and schistosomiasis; and (ii) to explore new strategies, with an emphasis on improved and more sensitive diagnostics and antimalarial therapies in elimination programmes.

## China: moving from disease control to elimination

Before 1955, just after the founding of the People’s Republic of China, many infectious diseases were highly endemic, particularly in the southern parts of the country, similar to the epidemic and pandemic documented in Africa between 1980s and 2000 [19]. The devastating impact of schistosomiasis is made explicit by the line in one of the poems by Chairman Mao Zedong: “Hundreds of villages overgrown with weeds, sick people shitting and thousands of desolate house-ghosts singing”, written in 1958. The epidemiological characteristics of schistosomiasis transmission demanded a well-structured and effective control programme put in place long before the new phase of social and economic development could gain foothold. By the end of 2011, 130 out of the initially 454 endemic counties in 12 provinces reached the criteria of transmission control and 274 had interrupted transmission completely. In parallel, the number of individuals infected with *Schistosoma japonicum* had declined to an all-time low with less than 300,000 infected people [23,24].

With regard to malaria, it must be noted that sustained efforts have reined in the endemic areas, which are now considerably smaller with the number of cases in China decreasing from an estimated 30 million in the 1940s to less than 5,000 by the end of 2011 [25]. Today, *Plasmodium falciparum* malaria has been eliminated as a public health threat in all provinces except Yunnan. Among the 24 malaria-endemic provinces, annual incidence rates have dropped below 1 per 10,000 in 95% of the endemic counties, and only 87 counties have a rate above this threshold [4,26,27]. Hence, in 2010, the Chinese Government decided to embark on malaria elimination by 2015, with the exception of Yunnan province, and to completely interrupt transmission across the country by 2020. Progress is monitored through a dynamic infectious disease reporting system that rigorously conducts case detection, collects and manages information covering the entire country, including all townships and counties where malaria is, or was, endemic [27-30].

Knowledge on the unrelenting control of its many endemic tropical diseases and the growing expertise in other disease-endemic regions of the world has shown that China can harness evidence-based knowledge and information, create innovative approaches and implement large-scale, effective solution for both its own people and the global community. The low-level transmission patterns of infection and re-infection rates in China and other endemic countries underscore that continuing control activities are critical for ultimate success of schistosomiasis and malaria surveillance-response systems. For example, transmission features in schistosomiasis include environmental contamination by eggs excreted by a large number of domestic and wild animal species that contribute to transmission; indeed the proportion of eggs from water buffaloes might account for up to 90% of all eggs found in the endemic areas [31,32]. In addition, transmission interruption of schistosomiasis would be marginal without this contribution. Hence, the current schistosomiasis control strategy in China places particular emphasis on the mechanisation of agriculture as this would reduce the number of water buffaloes substantially [33,34]. Evaluation of the new integrated control strategy carried out in 26 villages in six townships of four counties in four provinces (including the use of mollusciciding and innovative educational methods, geospatial mapping of hotspots, reservoir control through larval source management using larvicides, environmental management, snail control and mass drug administration (MDA) using praziquantel) curbed the human infection to 0-1% after three transmission seasons post-intervention. After four transmission seasons post-intervention, the intermediate host snail infection rate and the infection rate in sentinel mice were reduced to zero. Long-term monitoring has shown that it is possible to sustain the effectiveness of this strategy [5,32,35].

## Active case prevention and management with regard to elimination

It was documented that active mapping of disease transmission hotspots and molecular characterization of genetic diversity and population dynamics were effective for early detection, allowing prompt treatment using safe and efficacious drugs. Meanwhile, patient compliance and adherence to any effective prescribed regimen is vital for public health management in primary healthcare and minimising the evolution of resistant parasite strains resulting from sub-therapeutic doses and drug selective pressure indicators. There is a pressing need to develop new surveillance tools and strategic responses to shift the focus from control to elimination. With regard to local elimination of malaria in China, specific response packages must be tailored for different settings [36]. For example, malaria in Hainan province is characterised by imported cases whereas in Hunan province and along the border between Yunnan province and Myanmar, active *P. vivax* transmission and mixed infections with *P. falciparum* and *P. vivax* are documented, respectively [36,37].

Highly sensitive and specific diagnostic tools (e.g. simple, inexpensive and field-applicable molecular detection devices) are urgently needed to target mass screening of asymptomatic gametocyte reservoirs in low/moderate endemic areas as well as sub-microscopic parasitaemia and intensive integrated management of hotspots linked to environmental, climatic and ecological appropriateness for *Anopheles* vectors and transmission [36,38]. Intensification of the use of intermittent preventive treatment (IPT) in high-risk groups (e.g. young children and pregnant women in endemic-prone areas) and rigorous malaria surveillance-response systems are necessary for effective case management and follow-up. A policy for imported cases should be developed and implemented nationwide, placing particular emphasis on the local context and trans-boundary migrant populations [39]. Additionally, research on vector control in disease elimination was recommended, particularly integrated vector management and the development of new insecticides with low toxicity to humans and the environment. Furthermore, additional funding reinvigorating transmission-blocking vaccines targeting the liver and/or gametocyte stages of the parasite would be important [17,18].

New reliable tools and efficient strategies against other communicable diseases were discussed, such as development of highly sensitive diagnostic assays for early detection of low-transmission settings, MDA, development and validation of vaccines for prevention and drugs for management, improvement of preventive strategies and environmental sanitation, information, education and communication (IEC), community participation and ownership, mechanisation of agriculture, sewage drainage, building of public latrines, waste management and provision of clean water as well as modelling based on a minimal essential database approach (Table 1).

**Table 1:** Intervention tools for the elimination of targeted diseases

**Intervention tools**	**Targeted diseases**
Mass drug administration (MDA)	Malaria, schistosomiasis, lymphatic filariasis and other NTDs
Information, education and communication (IEC)	Malaria, schistosomiasis, lymphatic filariasis and other NTDs
Community participation and ownership	Malaria, schistosomiasis, lymphatic filariasis and other NTDs
Development of highly sensitive diagnostic assays for early detection of low transmission settings	Malaria, schistosomiasis, lymphatic filariasis and other NTDs
Mechanisation of agriculture	Schistosomiasis and other NTDs
Development and validation of vaccines for prevention and drugs for prevention and management	Malaria, schistosomiasis, lymphatic filariasis and other NTDs
Improvement of preventive strategies and environmental sanitation	Schistosomiasis and other NTDs
Sewage drainage	Schistosomiasis and other NTDs
Public latrines	Schistosomiasis and other NTDs
Waste management and provision of clean water	Malaria, schistosomiasis, lymphatic filariasis and other NTDs
Mathematical modelling using a minimal essential database approach	Malaria, schistosomiasis, lymphatic filariasis and other NTDs

## Implementation of Health in surveillance-response systems

Health system and health policy makers, and government will require development and implementation of new surveillance-response system policies in the context of infectious diseases elimination towards eradication (e.g. containment of private-sector workers importing parasitic diseases). New policies will include prevention and control, community-based mobilisation, IEC as well as participation and cooperation to re-enforce management, community ownership and development, such as mechanisation of agriculture and food production. For example, in malaria information system, remote sensing, web-based information, eHealth (healthcare supported by electronic management and communication) and mHealth (mobile health, that has emerged as a sub-segment of eHealth, the use of information and communication technology (ICT) for health services, information and communication) have proved useful in controlling malaria in remote areas and enhancing active surveillance as well as new and fast ways of gathering geographical, spatial, ecological and meteorological data relevant for the understanding of foci of vector transmission, dynamics mapping for patient follow-up, containment of infections or outbreaks and monitoring resistance [40-43].

In schistosomiasis, veterinary public health and emphasis on the socioeconomic importance of zoonoses are needed along with health education with special reference to farming and the development of a new policy for surveillance-response systems to contain outbreaks and resurgence. In addition to prevention, control and spatio-temporal surveillance tools relevant to the understanding of schistosomiasis transmission dynamics, mapping of infectivity and close monitoring of outbreaks are important [36,44-46].

## Determinants of surveillance-response systems in infectious diseases elimination

Surveillance and response represent the final, crucial steps in achieving effective elimination of a disease (i.e. interruption of local transmission), as recognised in many of the ongoing malaria and NTD elimination programmes [47-49]. Introducing surveillance-response approaches characterise a paradigm shift in maintaining comprehensive monitoring and evaluation (M&E) activities towards focusing on the approaches that rapidly detect the remaining or re-emerging pockets or hotspots of transmission and allow swift public health action with adapted, integrated response packages to interrupt transmission. The key feature of effective surveillance-response systems should be based on the concept of collecting a minimal essential set of data to identify pockets of transmission, finding areas prone for re-emergence, establishment of a strategic role in long-term certification of elimination as well as an eye on potential resurgence of new cases and/or outbreaks. Hence, surveillance-response systems are distinctively different from “classical” M&E that aims at collecting all possible data/indicators that often leads to information overflow and little or delayed feedback of programme control operations to elimination programmes management, and hence absence of rapid effective public health action.

Passive and active surveillance systems have been utilised for different purposes and at different stages of disease control and elimination (Table 2). Passive surveillance often gathers data from all available reporting systems, aiming at reducing the public health burden in areas of high endemicity. The information from passive surveillance is used for setting sentinel sites for parasites, vectors and intermediate hosts, resistance monitoring, defining the geographical distribution of parasitic diseases and evaluating or synthesising the control or elimination programmes to respond to a particular target, for instance, M&E of the Global Fund to Fight AIDS, Tuberculosis and Malaria (http://www.theglobalfund.org/en/) [50,51]. Active surveillance places special emphasis on finding cases in the community mainly through door-to-door surveys, or gathering of information from institutions and healthcare providers. For example, to identify immigrants infected with *P. falciparum*, a dry-season, breeding-site mapping and an epidemiological survey were conducted, followed by diagnosis of malaria patients in the community and treatment in combination with integrated vector management (e.g. indoor residual spraying (IRS) and long-lasting insecticidal net (LLIN) campaigns). In addition, in their move towards elimination of the vicious cycle of poverty and infectious diseases, malaria consortia should continuously provide technical assistance for health system strengthening and the development of innovative tools to improve malaria surveillance and provide national and district staff with the information they need to respond to malaria outbreaks as well as to individual cases [50,52-54].

**Table 2:** Key definitions of some common terms

**Term**	**Definition**
Classical definition of surveillance	Ongoing systematic collection, analysis and interpretation of data, usually incidence of cases of disease. In the WHO Global Malaria Action Plan (GMAP), surveillance is defined as follows: “.... is aimed at discovery, investigation, and elimination of continuing transmission, the prevention and cure of infection and final substantiation of claimed eradication.”
Surveillance and response or surveillance as an intervention	Aiming to reduce transmission through monitoring and evaluation (M&E), this activity requires a shift from measuring morbidity and mortality to detecting infections and measuring transmission using novel, field-ready tools and strategies for the active detection of asymptomatic infection. It may include DNA-based and/or serological biomarkers, new effective approaches tracking population dynamics, effective field-based mapping linked to databases, improved measurements of mapping transmission and improve the feasibility, efficiency and cost-effectiveness of new health information systems.
Active surveillance	A structure engaging health professional to frequently monitor health care providers or the population to gather information about health situations. Although not cost-effective, active surveillance provides the most accurate and timely information.
Passive surveillance	A structure by which a health authority takes delivery of reports submitted from district to provincial hospitals, clinics, public health units or other sources. It is a cost-effective approach covering most populations, and offering critical information for monitoring a community’s health. However, due to its dependency on data provision from different institutions or hospitals, the quality of data can be hard to assess.
Sentinel surveillance	A structure supported by a reporting system based on selected institutions or individuals that provide regular, complete reports on one or more diseases occurring ideally in a defined catchment. It also provides additional data on individual cases.
Integrated surveillance	A combination of active and passive systems using a single organisation that collects information about multiple diseases or behaviours of interest to several intervention programmes (e.g. health facility-based system may continuously gather data that are linked to disease-specific data and quality control of the information or on multiple infectious diseases and disorders.
Syndrome surveillance	An active or passive system that uses case definitions that are based entirely on clinical features without any clinical or laboratory diagnosis. It is inexpensive and is faster than systems that require laboratory confirmation mostly applied in developing countries but lack of specificity (subjective) and require further analysis.
Control	The reduction of disease incidence, prevalence, morbidity or mortality to a locally acceptable level as a result of deliberate efforts.
Elimination	The reduction to zero of the incidence of infection in a defined geographical area as a result of deliberate efforts and continued measures to prevent re-establishment of transmission are required.
Eradication	The permanent reduction to zero of the worldwide incidence of infection, as a result of time-bound, deliberate efforts. Once eradication has been achieved, intervention measures are no longer needed.

The Forum elaborated on a set of effective and essential features to achieve infectious disease elimination, such as (i) political stability and good governance; (ii) commitment to use local resources for elimination activities; (iii) worthy organisational and technical infrastructure coupled with qualified and committed personnel; (iv) adequate general health care services delivery; (v) making communities understand and support the control programmes; (vi) no major uncontrolled population movements; and (vii) *P. vivax* and *P. falciparum* malaria addressed based on WHO’s global roadmap.

## A paradigm shift towards elimination

The move from morbidity control to interruption of transmission and achievement of elimination requires novel, advanced technologies, either aimed at reducing the infection reservoir or at reducing the rate at which infections spread. This can be done by an innovative research portfolio that is expanded to include NTDs, zoonoses and other socioeconomic determinants in most endemic countries, particularly in sub-Saharan Africa. Important outcomes from the First Forum on Surveillance-Response System Leading to Tropical Diseases Elimination include foremost important lessons from previous outbreaks and resurgences of parasitic diseases in China and elsewhere; second, new surveillance-response platforms capable of further strengthening existing health systems at all levels; third, long-term commitment, which is crucial to achieve sustainability of the elimination agenda; fourth, political will, i.e. commitment to use local resources and implementation of innovative multi-sectoral, integrated approaches; fifth, improved diagnostic tools for early detection of infection and disease; and finally, but not the least, capacity-building, new partnerships and use of innovative technology to render existing surveillance-response systems more effective [17,19,23,29,32,36,46,50,52,55].

The following questions were raised: (i) Do we have, and are we using, the most effective surveillance tools (this is of particular relevance with regard to diagnostics)? (ii) Are we implementing the most effective surveillance-response packages tailored to a given transmission setting? (iii) Is it possible to innovate and incorporate additional surveillance measures to existent interventions? (iv) What are the contributions of private companies to health? (v) How can we be sure that we move in the right direction? The Forum promptly progressed to addressing these questions during different sessions.

## The way forward and recommendations

Participants felt that the Forum provided a stimulating environment for exchange of ideas and experiences giving an impetus for rapid progress towards surveillance and response systems in elimination of tropical diseases that impact on the health and well-being of disadvantaged and marginalised populations in the world. It was concluded that the Forum should be held every 2 years. Hence, at the time of writing the current report, planning for the June 2014 forum is intensifying. The following recommendations for strengthening research on surveillance-response systems for disease elimination were put forward.

### Establishment of a resource-sharing platform for effective surveillance-response systems

The Forum recognised that containing the emergence and spread of tropical diseases requires early alertness, active detection and diagnosis of its pathogenic cause. Concerted efforts and effective partnerships are needed to share the roles and responsibilities in defining powerful surveillance-response systems to mitigate the public health burden of tropical diseases thereby ensuring scalability and sustainability.

Setting up a genuine coordination and collaboration between Asia Pacific and Africa with regard to public health and control/elimination of malaria and NTDs was encouraged, emphasising surveillance-response systems that rely on an integrated and dynamic approach to risk management with ongoing research, data collection and real-time analysis, thereby driving evidence-informed knowledge translation into policy decisions and action with a feedback process to facilitate continuous advancement and adaptation.

Continuous scrutiny of all aspects of infectious diseases occurrence and spread, pertinent to effective control and elimination, requires systematic collection, collation, analysis, interpretation and dissemination of health data and the establishment of a resource-sharing platform for surveillance-response system between Asia Pacific and Africa. Reconvening the Forum every 2 years would provide an opportunity to discuss progress and challenges in implementing local, national or regional surveillance-response system for targeted infectious diseases, discoveries and developments among scientists, public health specialists, politicians and other stakeholders.

Importantly, documented limitations call for dispersion of technological advances in most developing countries of Asia Pacific and Africa, so that surveillance-response strategies and rapid diagnostic methods, tools and approaches can be improved. This requires continued innovation, validation and application of efficient, low-cost diagnostic, affordable novel therapeutic agents and vaccines [4,5].

### Development of new tools and strategies

Sensitive diagnostic tools based on molecular methods and advanced biotechnological assays need to be developed as surveillance tools for monitoring and verification of elimination of tropical diseases [19,39,52,56]. Clinical outcomes and standard operating procedures for efficacy monitoring and safety assessment of drugs against malaria and NTDs must be carefully re-evaluated and cut-offs determined. In addition, *in vitro* sensitivity limitations of currently deployed detection tools (e.g. microscopy and rapid diagnostic tests) have been documented, and hence, there is a need for improved tools in assessing susceptibility [19,56].

The Forum provided a unique opportunity to discuss surveillance-response systems as the final stage towards elimination of infectious diseases, especially NTDs, based on a “One World-One Health” perspective. Five research priorities were recommended: (i) dynamic detection and mapping of transmission, particularly low-level transmission, also using eHealth and mHealth; (ii) near real-time monitoring of population dynamics; (iii) modelling to establish minimal essential databases and indicators to be collected in space and time; (iv) design of effective response packages tailored to different transmission settings and levels; and (v) rigorous validation of approaches and response packages with regards to effectiveness within elimination programmes.

### Certification of surveillance-response systems

It was emphasised that regional strategies, innovative data management tools, inter-sectoral, multi-disease control approaches and research framework on control and elimination of infectious diseases of poverty must be strengthened [55]. Surveillance-response systems directed towards elimination of malaria, schistosomiasis and other selected NTDs should be focusing on research priorities, innovative mechanisms, including tool development, targeting verification and certification of elimination. The central role that diagnostic tools play in certification of disease elimination is imperative and rigorous surveillance tools and data management are supportive in improving public health surveillance and response systems for prevention and control of priority-set diseases at all levels of national health systems [57,58].

### Transfer of Chinese experiences of surveillance-response systems to endemic countries with limited resources

China can provide expert support on the establishment of diagnostic tools and techniques, in providing capacity building and training on tropical diseases to laboratory technicians, medical staff and disease control managers in Africa. Moreover, support in monitoring techniques and conducting collaborative research on all aspects of effective surveillance-response mechanisms can be provided depending on context, nature of endemicity. Assistance can be provided to African countries to complement their own electronic reporting system for infectious diseases (e.g. malaria, schistosomiasis and other selected NTDs), including continuous monitoring and evaluation of disease transmission dynamics through innovative Sino-Africa partnership. China had developed effective surveillance and early warning systems (EWS) for malaria and schistosomiasis based on electronic-based reporting systems [45,59] and these resources can be transferred to other endemic countries with limited resources. China has successfully contained malaria re-emergence in Anhui province since the 2006 update of the situation [60]. Lessons learnt facilitate monitoring of focal prevalence, while modelling of changing transmission factors and trends prediction produce better tactics as well as strategic evidence not only for policy support, but also for direction, planning and implementation of pilot surveillance -response systems sites with respect to control/elimination.

It is worth noting that China has made significant contributions in the discovery and development of drugs against malaria (e.g. artemisinins), schistosomiasis (e.g. artemisinins and mefloquine) and soil-transmitted helminthiasis (e.g. tribendimidine) [61-63]. The artemisinins have been re-discovered by Chinese scientists in the late 1970s and combination therapy based on this drug (ACT) is the recommended first-line treatment in most malaria-endemic areas where multi-drug resistant *P. falciparum* and *P. vivax* (chloroquine, mefloquine and sulphadoxine-pyrimethamine) exist. Drug resistance is a very important consideration in malaria control demanding the application of approaches, such as real-time molecular genotyping of sexual and asexual resistant biomarkers from asymptomatic and symptomatic population. This activity is paramount in endemic areas for the monitoring of emerging trends of susceptibility and drug resistance, which are key components of malaria control towards elimination.

Future global health involvements include the establishment of comprehensive sites for M&E and impact assessment in sentinel sites, focussing on three areas of possible research-action programmes: (i) characterising how demographic, epidemiological and environmental factors influence disease transmission by comprehensive comparison of population prevalence, capacity building in diagnostic ability, effects of integrated vector management between demonstration areas and control areas; (ii) socioeconomic factors and the status of prevailing health systems; and (iii) strategy and measurement assessment, including evaluating intervention effects of a related policy and strategy applied in the interventional areas and further exploration of the external validity of the findings from these areas.

The Forum reviewed the action plans of NTD control and elimination articulated in the WHO roadmap and China CDC, as well as the national schistosomiasis elimination programme for China (2009-2015), placing particular emphasis on surveillance-response systems. China’s challenges and needs in tropical disease control, and its experiences and lessons learnt necessitate national governments to build and implement surveillance-response systems to deal with tropical diseases, including imported cases and new outbreaks.

The first Forum provided a platform for exchange among participants from different countries and sectors who presented their principles and approaches from disease control to elimination. The reader should, however, be aware that the current report expresses the opinions of the individuals participating in the Forum and does not intend to be an exhaustive exploration of the subject matter or a representation of consensus.

## Review and conclusion

During the First Forum on Surveillance-Response System Leading to Tropical Diseases Elimination, a priority research agenda within a ‘One Health-One World’ frame of global health was developed, including: (i) the establishment of a platform for resource-sharing and effective surveillance-response systems for Asia Pacific and Africa with an initial focus on elimination of lymphatic filariasis, malaria and schistosomiasis; (ii) development of new strategies, tools and approaches, such as improved diagnostics and antimalarial therapies; (iii) rigorous validation of surveillance-response systems; and (iv) designing pilot studies to transfer Chinese experiences of successful surveillance-response systems to endemic countries with limited resources.

More than 100 scientists, disease control managers and other stakeholders participated in the First Forum on Surveillance-Response System Leading to Tropical Diseases Elimination. It offered the opportunity for discussions and exchange of ideas and opinions with emphasis on disease elimination and the key role played by surveillance-response systems. Strengths and limitations of national and international strategies for elimination, prevailing strategies, and novel approaches required for elimination, rely on useful surveillance-response platforms. Funding channels should be explored to intensify research and development of new, innovative and effective platforms. Supporting Sino-Africa partnership for control of parasitic diseases will require taking initiatives for global health and the best experiences from China and the international community to Africa. New operational research should be devoted to identify risk factors and hotspots of transmission, monitoring emergence and spread of disease outbreak with enhanced geographical information systems (GIS), health systems, care management and an integrated approach in improving the efficiency of surveillance-response systems.

Sustained control can only be made leading to elimination by accelerating breakthroughs in research, while fully understanding inputs, processes, outcomes and impact on transmission. The launch of the WHO roadmap perpetuates the need for effective surveillance-response systems tailored to local settings. Systems effectiveness depends on access to highly sensitive diagnostics, prompt treatment and follow-up as well as quality of care and patient trust. Sensitive tools for surveillance for xenobiotic monitoring and a deeper understanding of which tools and strategies are most suitable to achieve elimination of malaria, schistosomiasis and other NTDs are needed. Moving from classical M&E to surveillance as an intervention and interruption of transmission not only requires high standards in data collection (minimal essential databases in space and time) and appropriate analysis, but also experiences in modelling and validation, verification of elimination and reliable forecasting. Finally, relevant effective intervention science is a must in order to improve the effectiveness of the policies recommended and the public health interventions undertaken.

## Competing interest

The authors declare that they have no competing interests.

## Authors’ contributions

ET, XZ, RB, MT, XNZ conceived the review concept. ET, LA, XZ, JHC, XNZ carried out the literature search, developed the structure for the manuscript, and drafted the paper. ET, XZ, RB, JGG, MT, XNZ participated to organize the draft sections, co-wrote sections of the draft, and ET, RB, JU, MT, XNZ edited the overall manuscript. All authors read and approved the final version of the manuscript before submission to Infectious Diseases of Poverty.

## Supplementary Material

Additional file 1 Multilingual abstracts in the six official working languages of the United Nations. Click here for file

## References

[B1] Utzinger J Raso G Brooker S de Savigny D Tanner M Ørnbjerg N Singer BH N’Goran EK Schistosomiasis and neglected tropical diseases: towards integrated and sustainable control and a word of caution Parasitology 2009 136 1859 1874 10.1017/S0031182009991600 19906318 PMC2791839

[B2] Li XX Zhou XN Co-infection of tuberculosis and parasitic diseases in humans: a systematic review Parasit Vectors 2013 6 79 10.1186/1756-3305-6-79 23522098 PMC3614457

[B3] Hotez PJ Molyneux DH Fenwick A Kumaresan J Ehrlich Sachs S Sachs JD Savioli L Control of neglected tropical diseases N Engl J Med 2007 357 1018 1027 10.1056/NEJMra064142 17804846

[B4] Chen JH Wang H Chen JX Bergquist R Tanner M Utzinger J Zhou XN Frontiers of parasitology research in the People’s Republic of China: infection, diagnosis, protection and surveillance Parasit Vectors 2012 5 221 10.1186/1756-3305-5-221 23036110 PMC3497869

[B5] Zhou XN Bergquist R Tanner M Elimination of tropical disease through surveillance and response Infect Dis Poverty 2013 2 1 10.1186/2049-9957-2-1 23849433 PMC3707090

[B6] Molyneux DH “Neglected” diseases but unrecognised successes – challenges and opportunities for infectious disease control Lancet 2004 364 380 383 10.1016/S0140-6736(04)16728-7 15276399

[B7] Hotez PJ Molyneux DH Fenwick A Ottesen E Ehrlich Sachs S Sachs JD Incorporating a rapid-impact package for neglected tropical diseases with programs for HIV/AIDS, tuberculosis, and malaria PLoS Med 2006 3 e102 10.1371/journal.pmed.0030102 16435908 PMC1351920

[B8] Utzinger J Becker SL Knopp S Blum J Neumayr AL Keiser J Hatz CF Neglected tropical diseases: diagnosis, clinical management, treatment and control Swiss Med Wkly 2012 142 w13727 23180107 10.4414/smw.2012.13727

[B9] Hotez PJ NTDs V.2.0: “blue marble health” -- neglected tropical disease control and elimination in a shifting health policy landscape PLoS Negl Trop Dis 2013 7 e2570 10.1371/journal.pntd.0002570 24278496 PMC3836998

[B10] Bhattacharya S Reflections on the eradication of smallpox Lancet 2010 375 1602 1603 10.1016/S0140-6736(10)60692-7 20458782

[B11] Larson HJ Ghinai I Lessons from polio eradication Nature 2011 473 446 447 10.1038/473446a 21614056

[B12] Smith Gueye C Sanders KC Galappaththy GN Rundi C Tobgay T Sovannaroth S Gao Q Surya A Thakur GD Baquilod M Lee WJ Bobogare A Deniyage SL Satimai W Taleo G Hung NM Cotter C Hsiang MS Vestergaard LS Gosling RD Active case detection for malaria elimination: a survey among Asia Pacific countries Malar J 2013 12 358 10.1186/1475-2875-12-358 24103345 PMC3852840

[B13] Murray CJL Vos T Lozano R Naghavi M Flaxman AD Michaud C Ezzati M Shibuya K Salomon JA Abdalla S Aboyans V Abraham J Ackerman I Aggarwal R Ahn SY Ali MK Alvarado M Anderson HR Anderson LM Andrews KG Atkinson C Baddour LM Bahalim AN Barker-Collo S Barrero LH Bartels DH Basáñez MG Baxter A Bell ML Benjamin EJ Disability-adjusted life years (DALYs) for 291 diseases and injuries in 21 regions, 1990-2010: a systematic analysis for the Global Burden of Disease study 2010 Lancet 2012 380 2197 2223 10.1016/S0140-6736(12)61689-4 23245608

[B14] WHO Accelerating work to overcome the global impact of neglected tropical diseases – a roadmap for implementation 2012 Geneva: World Health Organization

[B15] Morse SS Mazet JA Woolhouse M Parrish CR Carroll D Karesh WB Zambrana-Torrelio C Lipkin WI Daszak P Prediction and prevention of the next pandemic zoonosis Lancet 2012 380 1956 1965 10.1016/S0140-6736(12)61684-5 23200504 PMC3712877

[B16] Zhang H Lai S Wang L Zhao D Zhou D Lan Y Buckeridge DL Li Z Yang W Improving the performance of outbreak detection algorithms by classifying the levels of disease incidence PLoS One 2013 8 e71803 10.1371/journal.pone.0071803 23977146 PMC3747136

[B17] Moonen B Cohen JM Snow RW Slutsker L Drakeley C Smith DL Abeyasinghe RR Rodriguez MH Maharaj R Tanner M Targett G Operational strategies to achieve and maintain malaria elimination Lancet 2010 376 1592 1603 10.1016/S0140-6736(10)61269-X 21035841 PMC3037542

[B18] Bousema T Griffin JT Sauerwein RW Smith DL Churcher TS Takken W Ghani A Drakley C Gosling RD Hitting hotspots: spatial targeting of malaria for control and elimination PLoS Med 2012 9 e1001165 10.1371/journal.pmed.1001165 22303287 PMC3269430

[B19] Tambo E Adedeji AA Huang F Chen JH Zhou SS Tang LH Scaling up impact of malaria control programmes: a tale of events in sub-Saharan Africa and People’s Republic of China Infect Dis Poverty 2012 1 7 10.1186/2049-9957-1-7 23849299 PMC3710198

[B20] Tanner MA Gray JJ Haaga DA Association of cotherapy supervision with client outcomes, attrition, and trainee effectiveness in a psychotherapy training clinic J Clin Psychol 2012 68 1241 1252 10.1002/jclp.21902 22899235

[B21] Utzinger J A research and development agenda for the control and elimination of human helminthiases PLoS Negl Trop Dis 2012 6 e1646 10.1371/journal.pntd.0001646 22545174 PMC3335882

[B22] Mwangoka G Ogutu B Msambichaka B Mzee T Salim N Kafuruki S Mpina M Shekalaghe S Tanner M Abdulla S Experience and challenges from clinical trials with malaria vaccines in Africa Malar J 2013 12 86 10.1186/1475-2875-12-86 23496910 PMC3599886

[B23] Qian YJ Li SZ Xu J Yang K Huang YX Cao ZG Miu F Dang H Zhang LJ Zhang L Wang Q Bergquist R Zhou XN Potential schistosomiasis foci in China: a prospective study for schistosomiasis surveillance and response Acta Trop 2013 in press; doi:10.1016/j.actatropica.2013.08.017 10.1016/j.actatropica.2013.08.017 24012536

[B24] Xu J Xu JF Li SZ Zhang LJ Wang Q Zhu HH Zhou XN Integrated control programmes for schistosomiasis and other helminth infections in P.R. China Acta Trop 2013 in press; doi:10.1016/j.actatropica.2013.11.028 10.1016/j.actatropica.2013.11.028 24361182

[B25] Zheng Q Vanderslott S Jiang B Xu LL Liu CS Huo LL Duan LP Wu NB Li SZ Xia ZG Wu WP Hu W Zhang HB Research gaps for three main tropical diseases in the People’s Republic of China Infect Dis Poverty 2013 2 15 10.1186/2049-9957-2-15 23895635 PMC3751495

[B26] Zhou SS Wang Y Xia ZG Malaria situation in the People’s Republic of China in 2009 Chin J Parasitol Parasit Dis 2011 29 1 3 (in Chinese) 21823314

[B27] Xia ZG Yang MN Zhou SS Malaria situation in the People’s Republic of China in 2011 Chin J Parasitol Parasit Dis 2012 30 419 422 (in Chinese) 23484249

[B28] Hsiang MS Hwang J Kunene S Drakeley C Kandula D Novotny JM Parizo J Jensen T Tong M Kemere J Dlamini S Moonen B Angov E Dutta S Ockenhouse C Dorsey G Greenhouse B Surveillance for malaria elimination in Swaziland: a national cross-sectional study using pooled PCR and serology PLoS One 2012 7 e29550 10.1371/journal.pone.0029550 22238621 PMC3253098

[B29] Zhou SS Zhang SS Wang JJ Zheng X Huang F Li WD Xu X Zhang HW Spatial correlation between malaria cases and water-bodies in *Anopheles sinensis* dominated areas of Huang-Huai plain, China Parasit Vectors 2012 5 106 10.1186/1756-3305-5-106 22650153 PMC3414776

[B30] Yin JH Yang MN Zhou SS Wang Y Feng J Xia ZG Changing malaria transmission and implications in China towards national malaria elimination programme between 2010 and 2012 PLoS One 2013 8 e74228 10.1371/journal.pone.0074228 24040210 PMC3767829

[B31] Wu XH Wang XH Utzinger J Yang K Kristensen TK Bergsquist R Zhao GM Dang H Zhou XN Spatio-temporal correlation between human and bovine schistosomiasis in China: insight from three national sampling surveys Geospat Health 2007 2 75 84 18686257 10.4081/gh.2007.256

[B32] Wang LD Guo JG Wu XH Chen HG Wang TP Zhu SP Zhang ZH Steinmann P Yang GJ Wang SP Wu ZD Wang LY Hao Y Bergquist R Utzinger J Zhou XN China’s new strategy to block *Schistosoma japonicum* transmission: experiences and impact beyond schistosomiasis Trop Med Int Health 2009 14 1475 1483 10.1111/j.1365-3156.2009.02403.x 19793080

[B33] Wang LD Chen HG Guo JG Zeng XJ Hong XL Xiong JJ Wu XH Wang XH Wang LY Xia G Hao Y Chin DP Zhou XN A strategy to control transmission of *Schistosoma japonicum* in China N Engl J Med 2009 360 121 128 10.1056/NEJMoa0800135 19129526

[B34] McWilliam HE Piedrafita D Li Y Zheng M He Y Yu X McManus DP Meeusen EN Local immune responses of the Chinese water buffalo, *Bubalus bubalis*, against *Schistosoma japonicum* larvae: crucial insights for vaccine design PLoS Negl Trop Dis 2013 7 e2460 10.1371/journal.pntd.0002460 24086786 PMC3784499

[B35] Utzinger J Bergquist R Olveda R Zhou XN Important helminth infections in Southeast Asia: diversity, potential for control and prospects for elimination Adv Parasitol 2010 72 1 30 20624526 10.1016/S0065-308X(10)72001-7

[B36] Yang GJ Tanner M Utzinger J Malone JB Bergquist R Chan EY Gao Q Zhou XN Malaria surveillance-response strategies in different transmission zones of the People’s Republic of China: preparing for climate change Malar J 2012 11 426 10.1186/1475-2875-11-426 23256579 PMC3547762

[B37] Bi Y Hu W Yang H Zhou XN Yu W Guo Y Tong S Spatial patterns of malaria reported deaths in Yunnan province, China Am J Trop Med Hyg 2013 88 526 535 10.4269/ajtmh.2012.12-0217 23269660 PMC3592536

[B38] Barclay VC Smith RA Findeis JL Surveillance considerations for malaria elimination Malar J 2012 11 304 10.1186/1475-2875-11-304 22938625 PMC3480880

[B39] Zhou XN Prioritizing research for “One health - One world” Infect Dis Poverty 2012 1 1 10.1186/2049-9957-1-1 23849840 PMC3710101

[B40] Noor AM Mutheu JJ Tatem AJ Hay SI Snow RW Insecticide-treated net coverage in Africa: mapping progress in 2000-07 Lancet 2009 373 58 67 10.1016/S0140-6736(08)61596-2 19019422 PMC2652031

[B41] Gray DJ McManus DP Li Y Williams GM Bergquist R Ross AG Schistosomiasis elimination: lessons from the past guide the future Lancet Infect Dis 2010 10 733 736 10.1016/S1473-3099(10)70099-2 20705513

[B42] Bergquist R Whittaker M Control of neglected tropical diseases in Asia Pacific: implications for health information priorities Infect Dis Poverty 2012 1 3 10.1186/2049-9957-1-3 23849136 PMC3710194

[B43] Mwangangi JM Mbogo CM Orindi BO Muturi EJ Midega JT Nzovu J Gatakaa H Githure J Borgemeister C Keating J Beier JC Shifts in malaria vector species composition and transmission dynamics along the Kenyan coast over the past 20 years Malar J 2013 12 13 10.1186/1475-2875-12-13 23297732 PMC3544599

[B44] Hui FM Xu B Chen ZW Cheng X Liang L Huang HB Fang LQ Yang H Zhou HN Yang HL Zhou XN Cao WC Gong P Spatio-temporal distribution of malaria in Yunnan province, China Am J Trop Med Hyg 2009 81 503 509 19706922

[B45] Yang GJ Gao Q Zhou SS Malone JB McCarroll JC Tanner M Vounatsou P Bergquist R Utzinger J Zhou XN Mapping and predicting malaria transmission in the People’s Republic of China, using integrated biology-driven and statistical models Geospat Health 2010 5 11 22 21080317 10.4081/gh.2010.183

[B46] Kelly GC Tanner M Vallely A Clements A Malaria elimination: moving forward with spatial decision support systems Trends Parasitol 2012 28 297 304 10.1016/j.pt.2012.04.002 22607693

[B47] Alonso PL Brown G Arevalo-Herrera M Binka F Chitnis C Collins F Doumbo OK Greenwood B Hall BF Levine MM Mendis K Newman RD Plowe CV Rodríguez MH Sinden R Slutsker L Tanner M A research agenda to underpin malaria eradication PLoS Med 2011 8 e1000406 10.1371/journal.pmed.1000406 21311579 PMC3026687

[B48] malERA Consultative Group on Health Systems and Operational Research A research agenda for malaria eradication: health systems and operational research PLoS Med 2011 8 e1000397 21311588 10.1371/journal.pmed.1000397 PMC3026705

[B49] Hopkins DR Disease eradication N Engl J Med 2013 368 54 63 10.1056/NEJMra1200391 23281976

[B50] Kaneko A A community-directed strategy for sustainable malaria elimination on islands: short-term MDA integrated with ITNs and robust surveillance Acta Trop 2010 114 177 183 10.1016/j.actatropica.2010.01.012 20132788

[B51] Collins C Xu J Tang S Schistosomiasis control and the health system in P.R. China Infect Dis Poverty 2012 1 8 10.1186/2049-9957-1-8 23849320 PMC3710143

[B52] Atkinson JA Johnson ML Wijesinghe R Bobogare A Losi L O’Sullivan M Yamaguchi Y Kenilorea G Vallely A Cheng Q Ebringer A Bain L Gray K Harris I Whittaker M Reid H Clements A Shanks D Operational research to inform a sub-national surveillance intervention for malaria elimination in Solomon Islands Malar J 2012 11 101 10.1186/1475-2875-11-101 22462770 PMC3359162

[B53] So AD Ruiz-Esparza Q Technology innovation for infectious diseases in the developing world Infect Dis Poverty 2012 1 2 10.1186/2049-9957-1-2 23849080 PMC3710188

[B54] Zofou D Nyasa RB Nsagha DS Ntie-Kang F Meriki HD Assob JC Kuete V Control of malaria and other vector-borne protozoan diseases in the tropics: enduring challenges despite considerable progress and achievements Infect Dis Poverty 2014 3 1 10.1186/2049-9957-3-1 24401663 PMC3895778

[B55] Nakagawa J Ehrenberg JP Nealon J Fürst T Aratchige P Gonzales G Chanthavisouk C Hernandez LM Fengthong T Utzinger J Steinmann P Towards effective prevention and control of helminth neglected tropical diseases in the Western Pacific Region through multi-disease and multi-sectoral interventions Acta Trop 2013 in press; doi:10.1016/j.actatropica.2013.05.010 10.1016/j.actatropica.2013.05.010 23792012

[B56] Tan CG Sandhu HS Crawford DC Redd SC Beach MJ Buehler JW Bresnitz EA Pinner RW Bell BP Regional Anthrax Surveillance Team Surveillance for anthrax cases associated with contaminated letters, New Jersey, Delaware, and Pennsylvania, 2001 Emerg Inf Dis 2002 8 1073 1077 10.3201/eid0810.020322 PMC2730289 12396918

[B57] Bergquist R Johansen MV Utzinger J Diagnostic dilemmas in helminthology: what tools to use and when? Trends Parasitol 2009 25 151 156 10.1016/j.pt.2009.01.004 19269899

[B58] Huntington D Health systems perspectives - infectious diseases of poverty Infect Dis Poverty 2012 1 12 10.1186/2049-9957-1-12 23848993 PMC3710094

[B59] Wang WM Zhou HY Liu YB Li JL Cao YY Cao J Establishment of malaria early warning system in Jiangsu province: II. Application of digital Earth system in malaria epidemic management and surveillance Chin J Schisto Contr 2013 25 172 176 (in Chinese) 23894839

[B60] Zhu G Xia H Zhou H Li J Lu F Liu Y Cao J Gao Q Sattabongkot J Susceptibility of *Anopheles sinensis* to *Plasmodium vivax* in malarial outbreak areas of central China Parasit Vectors 2013 6 176 10.1186/1756-3305-6-176 23768077 PMC3695883

[B61] White NJ Qinghaosu (artemisinin): the price of success Science 2008 320 330 334 10.1126/science.1155165 18420924

[B62] Xiao SH Keiser J Chen MG Tanner M Utzinger J Research and development of antischistosomal drugs in the People’s Republic of China: a 60-year review Adv Parasitol 2010 73 231 295 20627145 10.1016/S0065-308X(10)73009-8

[B63] Xiao SH Utzinger J Tanner M Keiser J Xue J Advances with the Chinese anthelminthic drug tribendimidine in clinical trials and laboratory investigations Acta Trop 2013 126 115 126 10.1016/j.actatropica.2013.01.009 23352956

