# Supplementary material for: Surveillance-response systems: the key to elimination of tropical diseases
Source: Infect Dis Poverty. 2014 May 27;3:17. doi: 10.1186/2049-9957-3-17 (PMC4071800; doi:10.1186/2049-9957-3-17)
Supplement: Additional file 1 — Multilingual abstracts in the six official working languages of the United Nations. [file 2049-9957-3-17-S1.pdf]

Translation of the abstract into the six official working languages of the United Nations

منظومات المراقبة والاستجابة: مفاتيح لاستئصال الأمراض المدارية.

إرنِست تامبو ، لِن آي ، زيا زْهو ، جُون-هو تشِن ، وِيي هُو ، روبرت بيرجكِيست ، جيا جانج جُوو ، جوورج أُوتزينجَر ، مارسِل تَنّر ، زياو-نونج زْهو.

ملخص.
الأمراض المدارية (أمراض المناطق الحارة) مازالت هي السبب الأكبر للحالات المرضية وللوفايات في البلاد النامية. وعلى الرغم من أنّ الجهود الصحّية المتضافرة أحدثت تحسّناً بارزاً على مدار العشرين سنة الماضية ، فإن المجتمعات ذات الأوضاع محدودة الموارد تفتقد وسائل تقوية بيئتها في الاتجاه الذي يقدم ظروفاً أقل مواتاة أو أقل إيجابية للكائنات المُمرضة. ومع ذلك ، فإن تأثير الأمراض المعدية قد بدأ في الهبوط على مستوى العالم ، سوياً مع التقدّم الذي تحقق فيما يتعلّق بالاستجابة للمشاكل الصحيّة الأساسية ، والتحسّن في تقديم الخدمات الصحية للسكّان الأكثر عرضة للمرض.

بيان لندن عن الأمراض المدارية المهمَلة NTD ، والذي بادرت به خارطة طريق: "الأمراض المدارية المهمَلة NTD" لمنظمة الصحة العالمية WHO ، وضع المسار للسيطرة على ، والاستئصال الفعلي لعدّة أمراض مدارية بحلول العام 2020 مقدّماً دافعاً للبرامج المحلّية والإقليمية لاستئصال الأمراض. والأمراض المدارية تكون غالباً تناثرية شاردة ، وتكون الأولويات حيالها متباينة في الأقطار محدودة الموارد والمتوطّنة على مستويات متنوّعة من منظومات الصحّة العامة فيها. وكيما يجري تحديد وترتيب أولوية البحث المنهاجي في استئصال الأمراض المدارية ، فقد انعقد "المنتدى الأوّل عن منظومة المراقبة والاستجابة المفضي إلى استئصال الأمراض المدارية في مدينة شانجهاي ، وذلك في شهر يونيو (حُزيران) من العام 2012. وقد استُعرضت في ذلك المنتدى المنهجيات السارية ، وخارطة طريق الأمراض المدارية المهمَلة NTD ، وتبع ذلك مناقشات حول كيفية تحديد واستبيان المعوقات والفرص السائدتين ، بما في ذلك التعاون بين القطاعات ، وسُبل الاستئصال لامراض عددة معاً.

وقد تم تطوير جدول أعمال لأولويات الأبحاث في إطار المفهوم: "صحّة واحدة لعالم واحد One Health-OneWorld" لصحة عالمية ، وشمل ذلك: (1) إقامة قاعدة لاقتسام الموارد ، ومنظومات مراقبة واستجابة فعّالة لأقطار غرب المحيط الهادئ Asia Pacific وأفريقيا بتركيز مبدئي على استئصال داء الخيطيات اللمفاوية (داء الفيل) ، والملاريا ، وداء المنشقات (البلهارسيا) ؛ (2) تطوير منهجيات جديدة ، وأدوات ، وسُبُل ، مثل التشخيصات المحسّنة ، والعلاجات المضادة للملاريا ؛ (3) تحقيق دقيقة جدّاً لمنظومات المراقبة والاستجابة ؛ (4) تصميم دراسات إرشادية أو دليلية لنقل الخبرات الصينية لمنظومات المراقبة والاستجابة الناجحة إلى الأقطار المتوطّنة ذات الموارد المحدودة.

Translated from English version into Arabic by Lotfi Abdolhaleem, through

监测响应系统：热带病消除的关键

Ernest Tambo，艾琳，周霞，陈军虎，胡薇，Robert Bergquist，郭家钢，Jürg Utzinger, Marcel Tanner，周晓农

摘要

热带病（NTD）仍是导致发展中国家高发病率和死亡率的主要原因。虽然在过去的 20 年中，卫生工作已有了显著改善，但在资源有限的社区仍需要加强对热带病的环境治理，从而使环境不适合病原体的生长。感染性疾病在世界范围内的影响已经开始下降，人们对基本健康问题和提供给弱势群体的卫生服务的改善也越来越受到关注。世界卫生组织 NTD 路径图推动了伦敦被忽视热带病宣言的发布，制定了到 2020 年控制和最终消除部分热带疾病的计划，促进了各地和各区域的疾病消灭提供规划。热带疾病往往有着散发且不稳定的特性，并在资源有限和流行国家的公共卫生体系中存在着不同的等级。为了对消除热带疾病确定和优化战略研究重点，第一届热带病消除的监测-响应系统会议于 2012 年 6 月在上海举行。会议对当前战略和 NTD 路线图进行了回顾，并研讨了如何认识和审视流行挑战和机遇，包括部门间的协作和几种传染性热带病消除的方法。制定了一个全球健康框架内的“同一个健康-同一个世界”的优先研究领域，包括建立:(i)资源共享和有效的亚太及非洲区域监测反应系统平台，首先着重在消除淋巴丝虫病、疟疾和血吸虫病；(ii)新的策略、工具和方法，如改良的诊断试剂和抗疟疾疗法；(iii) 监测响应系统的严格验证；(iv) 设计试验研究，使中国成功的监测响应系统的经验传输给资源有限的疾病流行国家。

Translated from English version into Chinese by Ai Lin, edited by Zhou Xiao-nong

Les systèmes de surveillance et d'intervention : élément clé pour l'élimination des maladies tropicales

Ernest Tambo, Lin Ai, Xia Zhou, Jun-Hu Chen, Wei Hu, Robert Bergquist, Jia-Gang Guo, Jürg Utzinger, Marcel Tanner, Xiao-Nong Zhou

Résumé

Les maladies tropicales restent une des causes principales de morbidité et de mortalité dans les pays en voie de développement. Malgré des améliorations significatives au cours des vingt dernières années grâce à une coordination des efforts sanitaires, dans les milieux où les ressources sont comptées, les communautés n'ont pas les moyens de renforcer leur environnement dans des directions qui offriraient des conditions moins favorables aux pathogènes. Malgré tout, l'impact des maladies infectieuses a commencé à décliner dans le monde entier, à mesure que progressait la réponse aux problèmes sanitaires de base et que s'améliorait l'offre de services sanitaires aux populations les plus vulnérables. La Déclaration de Londres sur les maladies tropicales négligées (MTN), initiée par le plan d'action sur les MTN de l'Organisation mondiale de la santé, a montré la voie vers le contrôle et, à terme, l'élimination de plusieurs maladies tropicales d'ici à 2020, en donnant un élan favorable aux programmes locaux et régionaux d'élimination des maladies. Les maladies tropicales se propagent souvent de manière imprévisible et irrégulière, et, dans les pays endémiques aux ressources limitées, les priorités diffèrent en fonction des niveaux de leur système de santé publique. Le premier Forum sur les systèmes de surveillance et d'intervention visant à éliminer les maladies tropicales s'est réuni à Shanghai en juin 2012 avec pour objectif d'identifier et de hiérarchiser la recherche stratégique sur l'élimination des maladies tropicales. Les stratégies actuelles et le plan d'action sur les MTN y ont été passés en revue, suivis de discussions sur la façon de déterminer et d'examiner d'un œil critique les plus grands défis et opportunités du moment, y compris la collaboration intersectorielle et les approches pluripathologiques dans l'élimination des maladies. Un agenda de recherche prioritaire y a été élaboré au sein d'un cadre de santé mondiale « Un monde, une santé », comprenant (i) l'établissement d'une plateforme pour le partage des ressources et pour des systèmes de surveillance et d'intervention efficaces pour l'Asie-Pacifique et l'Afrique, initialement axés sur l'élimination de la filariose lymphatique, le paludisme et la schistosomiase ; (ii) l'élaboration de nouvelles stratégies, de nouveaux outils et de nouvelles approches, tels que l'amélioration des

diagnostics et les traitements antipaludéens ; (iii) une validation rigoureuse des systèmes de surveillance et d'intervention ; et (iv) la conception d'études pilotes pour transférer les expériences chinoises de systèmes de surveillance et d'interventions réussis aux pays endémiques à ressources limitées.

Translated from English version into French by Acgiovan, through

TRANSLATORS WITHOUT BORDERS

Системы контроля и ответного реагирования: ключевые факторы устранения тропических болезней

Эрнест Тамбо, Лин Ай, Ксиа Жу, Юн-Ху Чен, Вей Ху, Роберт Бергквист, Джиа-Ганг Гуо, Юрг Утцингер, Марсель Таннер, Ксяо-Нонг Жу.

Реферат

Тропические болезни остаются основной причиной заболеваемости и смертности в развивающихся странах. Несмотря на то, что за последние 20 лет совместные усилия по охране здоровья привели к значительному улучшению ситуации, общины с ограниченными ресурсами не обладают возможностями принятия соответствующих мер по созданию условий, неблагоприятных для развития патогенных микроорганизмов или факторов. Тем не менее, воздействие инфекционных болезней начало сокращаться во всем мире наряду с успехами в решении основных проблем охраны здоровья и совершенствованием оказания услуг в области здравоохранения наиболее уязвимым представителям населения. Лондонская декларация по забытым тропическим болезням, инициированная перспективным планом Всемирной организации здравоохранения, наметила дорогу в направлении контроля и возможного устранения нескольких тропических болезней к 2020 году, и дала толчок к разработке местных и региональных программ по устранению болезней. Тропические болезни часто носят очаговый и неравномерный характер, и на разных уровнях систем общественного здравоохранения эндемичных стран, характеризующихся ограниченными ресурсами, присутствуют разные приоритеты. Для определения приоритетов стратегических исследований по устранению тропических болезней в июне 2012 года в Шанхае был созван 1й Форум по системе контроля и ответного реагирования, направленной на уничтожение тропических болезней. На форуме были обсуждены применяемые стратегии и перспективный план по забытым тропическим болезням, а также были рассмотрены проблемы определения и критического изучения преобладающих задач и возможностей, включая межотраслевое сотрудничество и многоцелевой подход к уничтожению болезней. Были определены приоритетные текущие исследовательские задачи в рамках программы «Один мир – одно здоровье», включая (i) создание платформы для разделения ресурсов и создания эффективных систем контроля и ответного реагирования для

Азиатско-тихоокеанского региона и Африки с начальным фокусом на устранение лимфатического филяриоза, малярии и шистомоза; (ii) развитие новых стратегий, инструментов и подходов, как, например, более совершенная диагностика и противомалярийная терапия; (iii) строгая проверка систем контроля и ответного реагирования; и (iv) создание пилотных исследований для переноса успешного китайского опыта по реализации систем контроля и ответного реагирования в эндемичные страны с ограниченными ресурсами.

Translated from English version into Russian by Alena Hrybouskaya, through

TRANSLATORS
WITHOUT BORDERS

Sistemas de vigilancia-respuesta: claves para la erradicación de las enfermedades tropicales

Ernest Tambo, Lin Ai, Xia Zhou, Jun-Hu Chen, Wei Hu, Robert Bergquist, Jia-Gang Guo, Jürg Utzinger, Marcel Tanner, Xiao-Nong Zhou

Resumen

Las enfermedades tropicales siguen siendo una de las causas principales de la morbilidad y la mortalidad en los países en vías de desarrollo. Si bien gracias a un trabajo conjunto en el ámbito de la salud se han logrado mejoras significativas en el transcurso de los últimos 20 años, las comunidades de zonas afectadas por una escasez de recursos no cuentan con los medios necesarios para fortalecer su entorno en direcciones que podrían generar condiciones menos propicias para los agentes patógenos. Aun así, el impacto de las enfermedades infecciosas ha comenzado a disminuir en todo el mundo y, al mismo tiempo, se está progresando con respecto a la respuesta a los problemas de salud básicos y en la mejora de los servicios de salud que se prestan a las poblaciones más vulnerables. La Declaración de Londres sobre las Enfermedades Tropicales Desatendidas (NTD, por sus siglas en inglés), una iniciativa de la Hoja de Ruta para NTD de la Organización Mundial de la Salud, abrió el camino para el control y la eventual erradicación de varias enfermedades tropicales para el año 2020, lo cual impulsa los programas de erradicación de enfermedades locales y regionales. Las enfermedades tropicales suelen ser aisladas y erráticas, y hay diferentes prioridades en los países endémicos y con recursos limitados en varios niveles de su sistema de salud pública. Para identificar y establecer prioridades sobre la investigación estratégica sobre la erradicación de enfermedades tropicales, se ha llevado a cabo el "1.er Foro sobre el sistema de vigilancia-respuesta que lleva a la erradicación de enfermedades tropicales" (*1st Forum on Surveillance-Response System Leading to Tropical Diseases Elimination*) en Shanghái en junio de 2012. Allí se revisaron las estrategias actuales y la hoja de ruta para NTD y luego se entablaron conversaciones acerca de cómo identificar y examinar de manera crítica los principales desafíos y oportunidades, incluida la colaboración intersectorial y los enfoques multi-enfermedad a la erradicación de enfermedades. Se desarrolló un programa de investigación dentro del marco global "*One Health-One World*" de salud global, el cual incluye (i) el establecimiento de una plataforma para compartir recursos y sistemas de vigilancia-respuesta eficaces para el Pacífico Asiático y África

con un foco inicial en la erradicación de la filariasis linfática, la malaria y la esquistosomiasis; (ii) el desarrollo de nuevas estrategias, herramientas y enfoques, tales como un mejor diagnóstico y tratamientos contra la malaria; (iii) la validación rigurosa de los sistemas de vigilancia-respuesta; y (iv) el diseño de estudios piloto para transferir las experiencias chinas de los sistemas de vigilancia-respuesta exitosas a los países endémicos con recursos limitados.

Translated from English version into Spanish by María Emilia Meini, through

TRANSLATORS WITHOUT BORDERS
